# Supplementary material for: Super-Resolution Imaging Reveals Dynamic Reticular Cytoophidia
Source: Int J Mol Sci. 2022 Oct 2;23(19):11698. doi: 10.3390/ijms231911698 (PMC9569780; doi:10.3390/ijms231911698)
Supplement: Supplementary file 1 [file ijms-23-11698-s001.zip › ijms-1905957-supplementary.pdf]

Super-resolution imaging reveals dynamic reticular cytoophidia

Yifan Fang, Yi-Lan Li, Xiao-Ming Li and Ji-Long Liu

SUPPLEMENTAL INFORMATION

SUPPLEMENTAL FIGURES S1 - S3

SUPPLEMENTAL TABLES S1 AND S2

SUPPLEMENTAL FIGURES S1 - S3 AND FIGURE LEGENDS

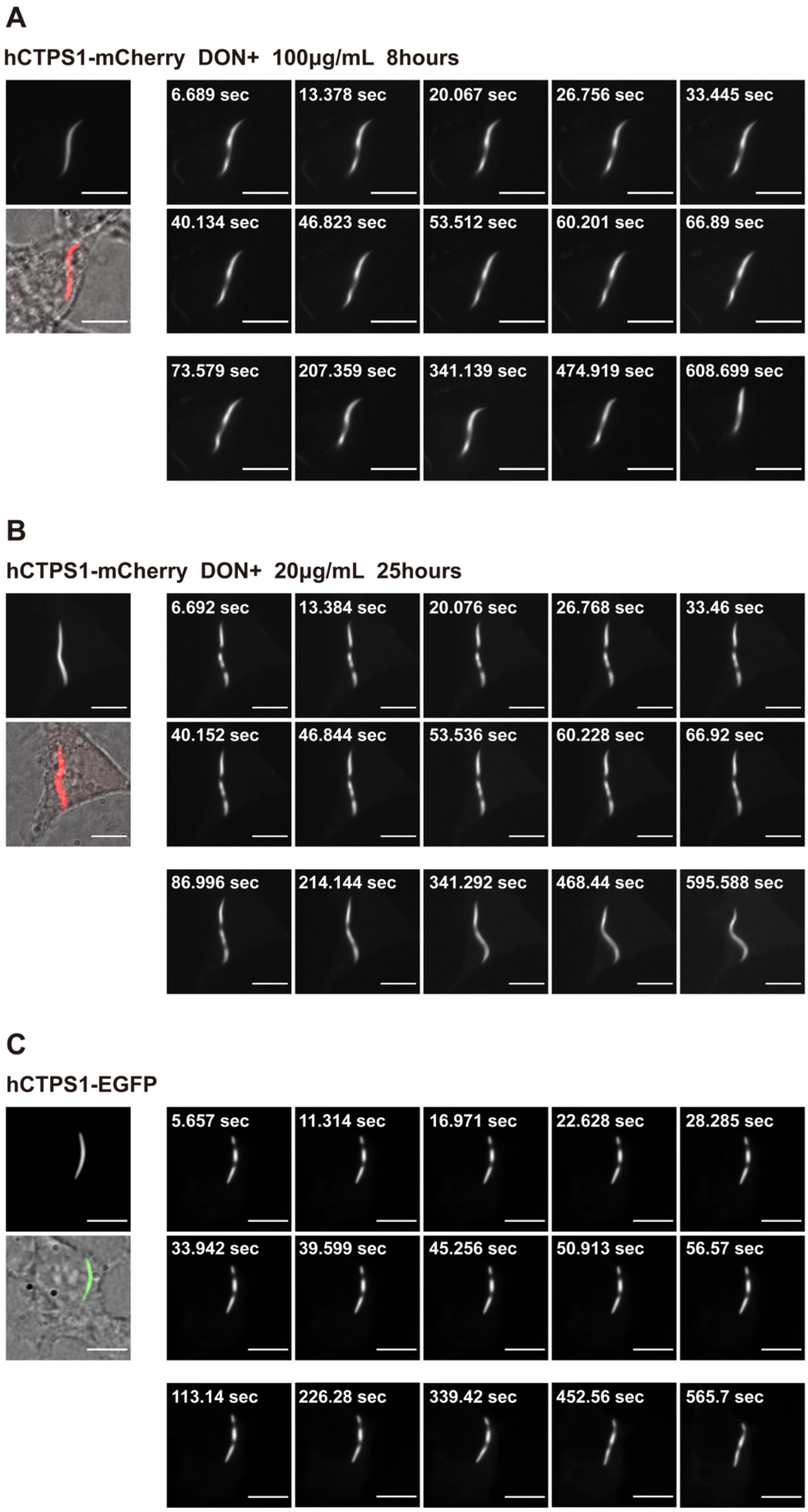

Figure S1. FRAP on cytoophidia.

(A) Living cell images of FRAP on hCTPS-mCherry cytoophidia induced by 100 μg/mL DON for 8 hours. (B) Living cell images of FRAP on hCTPS-mCherry cytoophidia induced by 20 μg/mL DON for 25 hours. (C) Living cell images of FRAP on hCTPS1-EGFP Cytoophidium-like condensates. Scale bars, 10 μm (A-C).

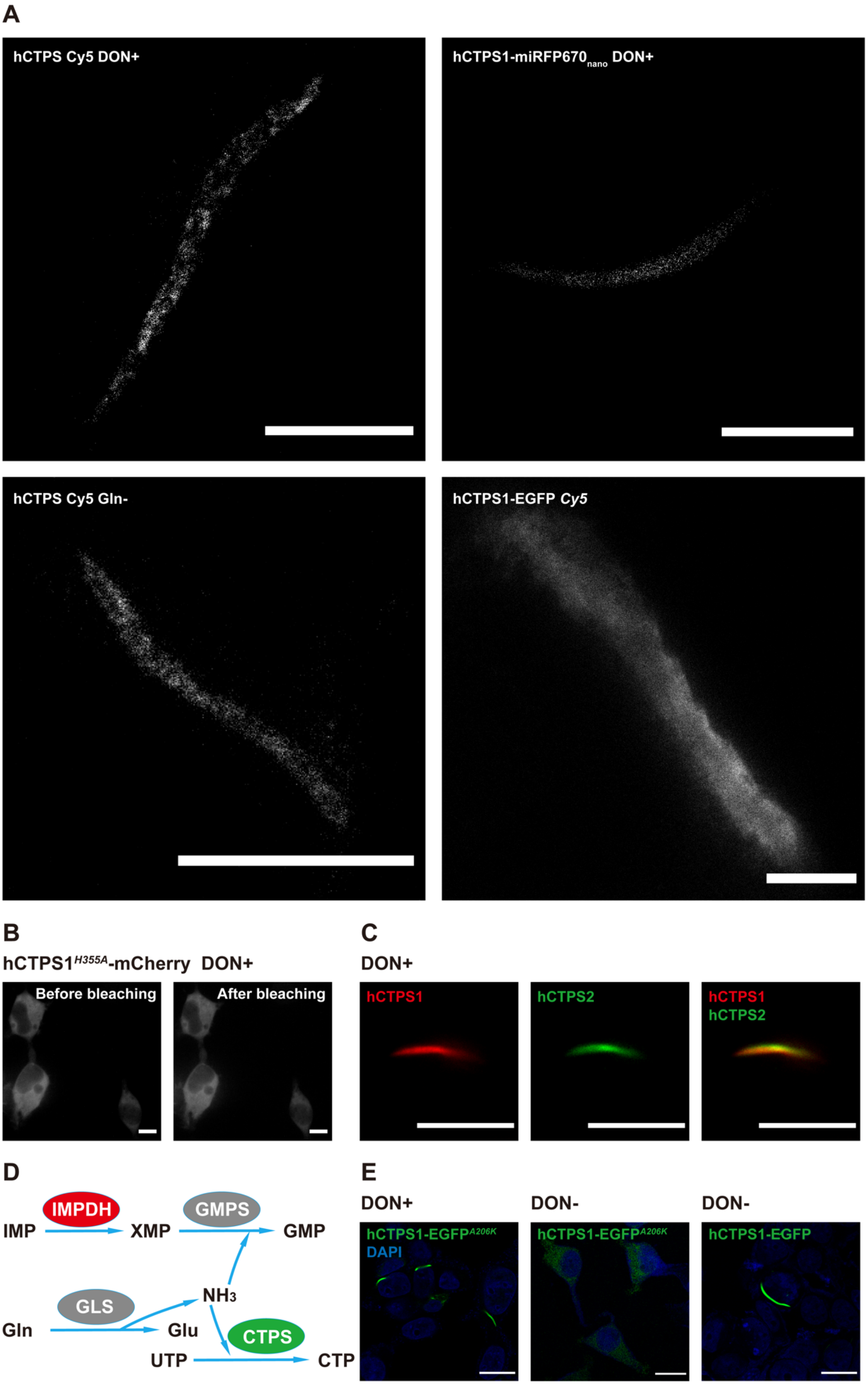
A
hCTPS Cy5 DON+
hCTPS1-miRFP670nano DON+
hCTPS Cy5 Gln-
hCTPS1-EGFP Cy5
B
hCTPS1H355A-mCherry DON+
Before bleaching
After bleaching
C
DON+
hCTPS1
hCTPS2
hCTPS1
hCTPS2
D
IMPDH
GMPS
IMP
XMP
GMP
NH3
GLS
Gln
Glu
CTPS
UTP
CTP
E
DON+
DON-
DON-
hCTPS1-EGFPA206K
DAPI
hCTPS1-EGFPA206K
hCTPS1-EGFP

Figure S2. Cytoophidia in super-resolution.

(A) STED images before deconvolution of 1) hCTPS cytoophidia induced by DON in 293T cells, 2) hCTPS1 cytoophidia with miRFP670nano tags induced by DON in living 293T cells, 3) hCTPS cytoophidia induced by glutamine deprivation in SW480 cells and 4) hCTPS1-EGFP cytoophidium-like condensates in 293T cells. (B) There was no difference in hCTPS1 signal before and after bleaching. (C) hCTPS1 and hCTPS2 were colocalized under DON treated in the form of cytoophidia. For DON treatment, DON (PBS solution) was added into fresh DMEM media 8 hours before living cell imaging. Images are not analyzed by SIM algorithm. (D) IMPDH and CTPS are both parts of NH_3 metabolic pathways. (E) hCTPS-EGFPA206K can form cytoophidia with DON treatment. Without DON, hCTPS-EGFPA206K cannot form cytoophidia. hCTPS1-EGFP can form cytoophidium-like condensates. For DON treatment, 20 μg/mL DON (PBS solution) was added into fresh DMEM media 8 hours before fixation. Scale bars, 3 μm (A), 10 μm (B, C) and 20 μm (E).

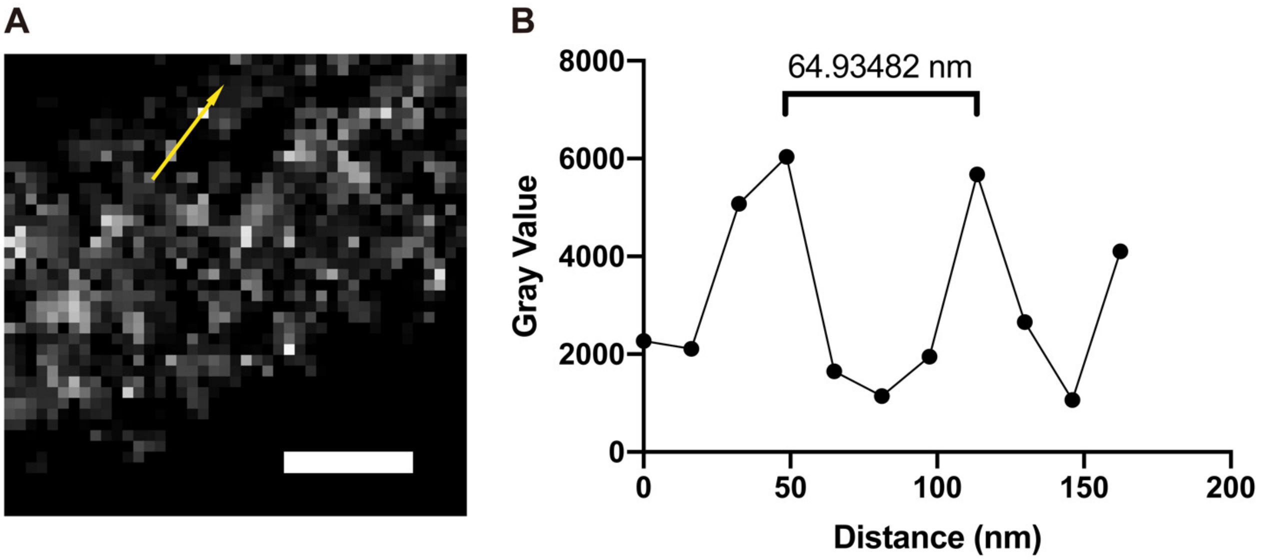

Figure S3. Resolution of STED microscopy.

(A) STED image zoomed in from figure 3A, hCTPS1-miRFP670nano cytoophidia induced by DON in live 293T cells, for resolution measurement along the arrow line. (B) By measuring intensity profiles along the lines marked in the image, the approximate resolution is 64.93482nm. Scale bars, 200 nm.

SUPPLEMENTAL TABLES S1 AND S2

Table S1. Over-expression proteins with fluorescence tags used in this study.

Exogenous proteins with tags	Figure
hCTPS1-mCherry	Figure 1A, B; Figure 2C; Figure 4A, C; Figure S1A, B; Figure S2B.
hCTPS1-EGFP^{A206K}	Figure 1H, Figure 3C, D.
hCTPS1-EGFP	Figure 1H; Figure S1C.
hCTPS1^{H355A}-EGFP	Figure 1H.
hCTPS1-miRFP670nano	Figure 3A, Figure S2A.
hCTPS2-miRFP670nano	Figure S2C.

Table S2. List of oligonucleotides used in this study.

Primer name	Sequence (5’-3’)	Target	Source
FYF-14F	ACGCGTTAAGTCGACAATCAACCTC	pLV-hCTPS1	This paper
FYF-14R	GTCATGATTTATTGATGGAAACTTCAGTTCGGTG	pLV-hCTPS1	This paper
FYF18-mCherry-F	TAAATCATGACCCACCGGTCATGGTGAGCAAGGGCGAGG	mCherry	This paper
FYF-13R	TGATTGTCGACTTAACGCGTTTACTTGTACAGCTCGTCCATGCCG	mCherry	This paper
FYF69-gfpK206A-F	GCCCTGAGCAAAGACCCCAACGAGAAGC	$EGFP^{K206A}$	This paper
FYF70-gfpK206A-R	GTCTTTGCTCAGGGCGGACTGGGTGCTCAGGTAGTGG	$EGFP^{K206A}$	This paper
FYF31F	GCAGAAGCTTGGCAGAAGCTCTGTAGTGC	$hCTPS1^{H355A}$	This paper
FYF32-R	CTGCCAAGCTTCTGCGTAGCGCACGGGCTCTTC	$hCTPS1^{H355A}$	This paper
FYF25-miRFPnano-FS	TAAATCATGACCCACCGGTCATGGCAAACCTGGACAAGATGCT	miRFP670nano	This paper

	GAA		
FYF28-miRFPnano-RS	TTGTCGACTTAACGCGTTTAGCTCTGCTGGATGGCGATGC	miRFP670nano	This paper
FYF50-S2-temp-R	CAGGATGTACTTCATGGTGGCAGCGCTCTAGAACCG	miRFP670nano-pLV	This paper
FYF51-S2-temp-F	GAGTTGGAAATAAGCCCACCGGTCATGGCAAACCTGG	miRFP670nano-pLV	This paper
FYF43-hCTPS2-F	ATGAAGTACATCCTGGTCACGGGTGG	hCTPS2	This paper
FYF44-hCTPS2-R	GCTTATTTCCAACTCAGCTATCCTTGGCTCT	hCTPS2	This paper
